# Resilience and distinct lifeways: sexual orientation and gender minority status differences in health, well-being, and social determinants of health in a population-based sample of older adults

**DOI:** 10.1186/s12889-025-25426-w

**Published:** 2025-12-23

**Authors:** Richard Bränström, Max Kleijberg, Anna Siverskog

**Affiliations:** 1 Division of Psychology, Department of Clinical Neuroscience, Karolinska Institutet, Nobels väg 9, Stockholm, 171 77 Sweden; 2 Department of Neurobiology, Care Sciences and Society, Karolinska Institutet, Stockholm, Sweden; 3 School of Innovation, Design and Engineering, Mälardalen University, Eskilstuna, Sweden; 4 Regional Cancer Centre Stockholm-Gotland, Stockholm, Sweden; 5 Department of Culture and Education, Södertörns högskola, Stockholm, Sweden

**Keywords:** LGBT aging, Minority stress, Social determinants of health, Resilience, Health disparities, Sweden

## Abstract

**Background:**

During the past two decades, differences in health and living conditions based on sexual orientation and gender minority status (e.g., those who identify as lesbian, gay, bisexual, and transgender) have received increased attention. The majority of these studies have been conducted among adolescents and young adults, and there is a need for more knowledge about the health situation among older generations of sexual and gender minority individuals. This study provides one of the most comprehensive population-based examinations of health disparities among older sexual orientation and gender minority adults outside the United States.

**Methods:**

This study explores disparities in health, well-being, and social determinants of health by sexual orientation and gender minority status in Sweden using data from 87,456 individuals aged 65 and older from the Swedish National Public Health Survey (2018–2022). We examined self-rated health, physical symptoms, psychological distress, and mental well-being, alongside social and economic health determinants such as discrimination, victimization, isolation, and financial stress.

**Results:**

While sexual and gender minority individuals reported higher levels of social and economic stressors, differences in health outcomes were modest. Gay men showed slightly higher psychological distress, and lesbian women reported lower positive well-being compared to heterosexual peers. There were no significant differences between cisgender and gender minority participants on any of our measures of health and well-being. Further, effect modification analyses showed that disparities were influenced by sociodemographic factors, with education level, migration background, and urbanicity acting as moderators.

**Conclusions:**

These findings suggest that despite elevated stressors, older sexual and gender minority adults may demonstrate resilience shaped by life experiences. Public health efforts should address structural inequalities while recognizing the strengths and diversity within this population.

**Supplementary Information:**

The online version contains supplementary material available at 10.1186/s12889-025-25426-w.

During the past two decades, differences in health and living conditions based on sexual orientation and gender minority status have received increased attention [1]. There is now a large body of research that clearly shows that sexual and gender minority (SGM) people, i.e., those with a minority sexual orientation and/or gender identity status (e.g. lesbian, gay, bisexual, and transgender people), have an increased risk of several stress-related health conditions, such as poor self-rated health [2], specific symptoms like asthma, allergy, and high blood pressure [2, 3], increased psychological distress [4–10], and other indicators of poor life-satisfaction [1], compared to heterosexual cis-gender people.

Epidemiological studies indicate that health disparities based on sexual orientation and gender minority status manifest early in life, with sexual and gender minority adolescents and young adults reporting poorer health outcomes compared to their heterosexual cisgender counterparts [11–14]. Studies of the age patterning of these disparities suggest that the disparities in mental health, although largest among young adults, continues into late adulthood [15, 16], whereas the disparities in physical health conditions and self-reported general health seems to be attenuated with increasing age [2]. However, since the majority of studies on sexual orientation and gender minority status disparities in health and well-being have been conducted among adolescents and young adults, more knowledge is needed to better understand the health situation among older generations of sexual and gender minority individuals [17]. Further arguments for the need of more research is that most people in older generations today grew up in an environment that was much more hostile to SGM individuals and studies have shown that acceptance and visibility of sexual and gender minority status have increased substantially in many parts of the world [18, 19]. The life situation for older SGM individuals might also differ in important ways from that of younger SGM individuals, e.g., a greater need for help from others, potentially lower ability to influence living arrangements, and a general decreased autonomy.

Until recently, models describing stigma and stigma-based social stress exposure, such as the minority stress theory [6] and the health equity promotion model [20], have been the most empirically supported models explaining the increased risk of poor health among SGM individuals. The minority stress theory has driven an extensive number of studies and enhanced our understanding of the role of stigma-based interpersonal stress in health disparities related primarily to sexual orientation [21], but the model has also been expanded to explain health disparities based on gender minority status [22, 23]. While the original model emphasizes the impact of distal and proximal stressors — such as discrimination, stigma, and internalized negativity — on marginalized groups, the gender minority model highlights additional stressors uniquely tied to gender identity. These include experiences of misgendering, barriers to accessing gender-affirming healthcare, and societal invalidation of non-cis-normative identities [24, 25]. By accounting for these distinct stressors, the gender minority model provides a more comprehensive understanding of the mental health disparities observed among gender-diverse individuals [26, 27].

Given that the majority of studies of determinants of sexual and gender minorities increased risk of poor health has been conducted among younger age groups and the fact that developmental challenges are known to vary across ages [28–31], the need to expand and further develop the minority stress framework has become increasingly important [32]. Recent research has identified additional determinants of the disproportionate health risks faced by sexual and gender minorities related to lack of societal integration and distinct lifeways [10, 31, 33]. These findings suggest new mechanisms that might have particular relevance for older sexual and gender minority populations. According to the recently proposed *distinct lifeways theory* [31], the lives of sexual minority individuals differ significantly from those of heterosexual cis-gender individuals affecting demographic and socioeconomic factors that might contribute to these groups’ health and well-being in both positive and negative ways. Sexual and gender minority individuals often occupy societal roles that differ from those of heterosexual cis-gender individuals in areas such as marriage/romantic partners, parenthood, friendships, and social networks [10, 31]. The consequences of these difference are believed to be potentially both positive and negative, e.g.: (a) by increasing risk of poor health and well-being (e.g., higher likelihood of living alone [10], being socially excluded [34]) and (b) by promoting health, well-being, and sense of meaning (e.g., strong community engagement [35], identity pride [36], support from a “chosen” family [37], feelings of authenticity [38]). Certain factors linked to sexual and gender minorities’ distinct lifeways, such as educational and occupational choices [39, 40], might also have consequences for well-established socioeconomic determinants of health (e.g., low household income, financial stress, and unemployment). While stigma related to sexual or gender minority identity may influence these societal roles and lifeways, they can also be stressful simply because they are less normative and less anticipated by a heteronormative society and the roles expected of heterosexual cis-gender individuals [31]. However, it should be noted that the distinct lifeways theory of sexual and gender minority health is currently not well explored [10, 31]. While it offers a promising framework, there is a clear need for more empirical studies. Greater knowledge is required to determine how, and in what ways, these lifeways actually influence the lived realities of sexual and gender minorities across diverse contexts.

With a few exceptions of studies in the US [41], most of the research on older sexual and gender minority populations’ health has been conducted in small and non-representative samples without a heterosexual cis-gender comparison group [35, 42–44]. It remains largely unexplored if older generations of sexual and gender minority individuals have an increased risk of poor health and well-being outside of the US. It is also unknown how social determinants of health linked to social stress and distinct lifeways are distributed among older sexual and gender minority individuals. To address these knowledge gaps and methodological limitations, we used a large, probability-based cross-sectional study of sexual minority and gender minority individuals aged 65 years and older with heterosexual cis-gender controls – to examine the following research questions: (a) Are sexual and gender minority older people more likely to report more physical health symptoms, psychological distress, and lower well-being compared to heterosexual cisgender people? (b) Are there sexual orientation and gender minority status differences in theory-derived determinants of health, i.e., interpersonal stress, social connectedness, and economic hardship, and (c) Are some sub-groups within the sexual and gender minority group particularly likely to experience poor health and well-being, based on: level of education (i.e., university degree vs. no university degree), migration background (i.e., Swedish born vs. non-Swedish born), geographic location (i.e., large city; medium/small town; rural area), and physical disability (i.e., self-reported disability vs. no self-reported disability)?

## Method

### Study sample

Each year in 2018, 2020, 2021, and 2022, random samples aged 16 and 84 years were drawn from population registries containing information about all residents in Sweden [45] and invited to complete a health survey - the Swedish National Public Health Survey. The survey is administered by the Public Health Agency of Sweden and questionnaires for each year can be assessed through their webpage (https://www.folkhalsomyndigheten.se/folkhalsorapportering-statistik/om-vara-datainsamlingar/nationella-folkhalsoenkaten/frageformular/). All 87,456 individuals 65 years-of-age and older who successfully returned the questionnaire and provided responses to the main variables in the current study were included in our total sample.

### Measures

#### Sexual orientation

 Individuals’ sexual orientation was categorized based on self-identification using the question: “What is your sexual orientation [heterosexual, lesbian/gay, bisexual, other, don’t know]?” Among those who reported an “other” sexual orientation only three respondents provided a free text sexual minority identity label. For analyses of groups differences participants were thus categorized as: (a) heterosexuals, (b) lesbian or gay, and (c) bisexual. Participants reporting that they don’t know their sexual orientation or reported another sexual minority identity than heterosexual, lesbian/gay, or pan-/bisexual were excluded from the analyses of sexual orientation difference (*n* = 4,811, 5.8%) but retained for the analyses of gender minority group differences.

#### Gender identity and gender minority status

 Respondents were asked, “How do you define your gender identity [woman, man, other]?” We used a categorization of gender modality [46], using information about gender identity, as well as, responses from the survey item: “Are you or have you been a trans person [yes/no]?” with a definition (“Trans person is a broad term that usually concerns individuals whose gender identity and/or gender expression sometimes or always differs from the norm of the gender they were assigned at birth.”). Respondents were categorized as gender minority if they reported either being a trans person or an “other” gender identity. The final categorization of gender modality were cisgender men, cisgender women, and gender minority.

#### Self-rated health status

 Self-rated health was assessed with the following item: “How would you rate your general health [very good, good, fair, poor, very poor]?” Consistent with prior research, we created a dichotomous variable comparing individuals with fair, poor, or very poor health versus those reporting good or very good health [47]. Prior research has demonstrated that self-rated health is a valid indicator of health status and/or the presence of disease and predicts mortality risk [48].

#### Physical health symptoms

 Number of physical symptoms was assessed with two items: “Do you currently have any of the following problems or symptoms [pain in neck, back pain, headache, pain in hand/arm/legs, fatigue, insomnia, dermatitis, tinnitus, urinary incontinence, and intestinal problems]?” and “Do you have any of the following conditions [diabetes, asthma, allergy, and high blood pressure]?” A count variable summarizing the experience of any of the 10 problems/symptoms and any of the four chronic physical condition was created, creating a variable that ranged from 0 to 14.

#### Psychological distress

 Psychological distress was measured with the 6-item Kessler psychological distress scale [49]. The scale consists of six items related to frequency of psychological distress symptoms (e.g., “worry,” “restlessness,” “hopelessness”) experienced using the past 30 days [50]. The scale has a total range from 0 to 24, with higher scores representing higher frequency of experienced symptoms of distress (Cronbach’s α = 0.89).

#### Positive mental health

 Positive mental health was assessed using the Warwick-Edinburgh Mental Well-being Scale (WEMWBS) [51]. The WEMWBS consists of seven items assessing frequency of positively phrased aspects of mental health (e.g., “feeling cheerful,” “feeling relaxed,” “interested in new things”) experienced during the past two weeks. The scale has a total range from 0 to 28, with higher scores representing greater experience of positive mental health (Cronbach’s α = 0.86).

#### Interpersonal stress

 Participants experience of *discrimination* during the past 3 months was assessed with the item: “During the past three months, have you been treated in a way that made you feel discriminated against [no, yes]?” Experience of victimization or threat during the past 12 months was assessed with the items: “During the past 12 months, have you been exposed to physical violence [no, yes]?” and “During the past 12 months, have you been exposed to a threat or threats of violence in a way that made you scared [no, yes]?” *Lack of social trust* was assessed with the question: “Do you think that people generally can rely on other people [no, yes]?” with participants being categorized as lacking social trust if they responded ‘no’ to the question.

#### Social connectedness

 Information on self-reported household composition “Who do you live with [no-one, parents or siblings, spouse or partner, other adults/-s, children]?”, was used to categorize participants as *living alone* or not. *Lack of social support* was assessed with two questions: “Do you have anyone you can share your innermost feeling with and confide in [no, yes]?” and “Can you get help from any person or persons if you have practical problems or are ill [no, yes]?” Participants were categorized as not having access to social support if they responded ‘no’ both questions. Low social participation was assessed with the survey item: “Have you taken part in any of the following activities in the past 12 months [list 13 social activities e.g., major family reunion, private party, sporting event, and none of the above]?” Respondents were categorized as low on social participation if they reported not having engaged in any of the 13 activities during the past 12 months.

#### Economic hardship

 Total disposable household income and self-reported financial stress were used as measures of economic hardship. Disposable household income for the year prior to the survey was collected from national registers kept by Statistics Sweden and linked to each participants’ survey responses based on the personal identification numbers available in Sweden. Participants were categorized as having low household income if they belonged to the lowest tertile of household income in the population. Self-reported financial stress was assessed using the item: “During the last 12 months, have you ever had difficulty in managing the regular expenses for food, rent, bills etc. [no, yes]?” and participants were categorized into those having experienced this or not during the past 12 months.

#### Variables used to analyze potential effect modification based on sociodemographic factors and physical disability

*Level of education* (“What is the highest grade of school you have completed?” with seven response alternatives ranging from “some high school” to “advanced graduate school degree”) where participants were categorized into those with or without a university degree, *migration background* (i.e., Swedish born vs. non-Swedish born), *geographic location* (i.e., large city; medium/small town; rural area), and *physical disability* (i.e., self-reported disability vs. no self-reported disability) was used to analyze potential effect modification, i.e., if sexual orientation differences varied at different levels of education, migration background, geographic location, and physical disability.

### Statistical analysis

After examining descriptive statistics of participants’ sociodemographic characteristics, we compared sexual orientation and gender identity differences in self-reported health, physical health symptoms, psychological distress, and positive mental health using Chi-2 test and Student’s *t*-test. Next, we descriptively compared difference in health outcome variables (i.e., poor self-rated health, physical health symptoms, psychological distress, and positive well-being) by sexual orientation for men and women separately, with separate estimates calculated for bisexual vs. heterosexual and lesbian/gay vs. heterosexuals. For these analyses, we used logistic regression analyses for binary outcomes and independent group t-tests for continuous and count variables. Odds ratios and Cohen’s *d* were used as measures of effect sizes. Similarly, we analyzed gender minority status differences in health outcomes with comparisons between gender minorities and cisgender respondents including all cisgender individuals irrespective of sexual orientation. Chi-2 tests were used to compare group differences in interpersonal stress, social connectedness, and economic hardship. Finally, we ran a series of interaction models to test potential effect modification. To conduct these analyses, we used moderated linear regression models to explore if group differences in our health outcomes varied significantly in sexual minority subgroups based on level of education, migration background, geographic location (i.e., large city; medium/small town; rural area), and physical disability.

To clarify any potential sub-group differences between sexual minority subgroups and potential differences between gender minority individuals compared to sexual minority individuals, we ran a series of supplementary analyses of sexual and gender minority sub-group differences on the main health and well-being outcome variables. These results are presented in a supplementary table (Supplementary Table S1).

Survey weights were used to adjust the sample to represent the national distribution based on Swedish census data. All analyses were conducted with SPSS version 26.

## Results

### Descriptive statistics

Tables 1 and 2 present the sociodemographic characteristics of the study sample with details reported separately by gender minority status (Tabel 1) and sexual orientation (Table 2). The most frequent sexual orientation in the sample was heterosexual (93.2%). Among those who reported a sexual minority identity, 57.3% identified as bisexual and 42.6% as lesbian or gay, and among those with “other sexual identity” (1.1%) the most frequently reported identity was an asexual identity. Gender minority respondents were more likely to have a university degree compared to cisgender respondents (*P* =.041), and gender minorities were also more likely to having been born outside of Sweden (*P* =.024) and to live in a larger city (*P* <.001). Sexual minority respondents were more than twice as likely to have a university degree (*P* =.001). Sexual minority respondents were also more likely to having been born outside of Sweden and to live in a larger city (*P* =.001).

**Table 1:** Sociodemographic distribution by gender modality in the Swedish National public health survey (2020, 2021, and 2022) among participants 65 years or older (n = 87,456)

	Total, *n* = 87,456		Cisgender men, *n* = 42,278		Cisgender women, *n* = 44,926		Gender minority, *n* = 252	
Mean age – years (SD)	75.1	(6.9)	75.0	(6.7)	75.1	(7.1)	75.0	(7.3)
Sexual orientation identity – *n* (%^a^)								
Heterosexual	81,881	(93.2)	39,410	(93.3)	42,275	(93.1)	196	(80.0)
Lesbian/gay	306	(0.4)	193	(0.6)	107	(0.3)	6	(2.9)
Bisexual	458	(0.6)	275	(0.7)	166	(0.5)	17	(5.7)
Other sexual orientation	922	(1.1)	494	(1.1)	421	(1.0)	7	(1.0)
I don’t know	3,889	(4.7)	1,906	(4.2)	1,957	(5.2)	26	(10.5)
Gender identity – n (% ^a^)								
Man	42,359	(48.6)	42,228	(100.0)	44,903	(100.0)	131	(57.7)
Woman	44,989	(51.4)	0	(0.0)	0	(0.0)	86	(28.8)
Non-binary or another gender identity	29	(0.04)	0	(0.0)	0	(0.0)	29	(13.5)
Transgender status – n (% ^a^)								
Cis-gender	86,018	(99.7)	7,437	(100.0)	8,994	(100.0)	10	(9.7)
Transgender	230	(0.3)	0	(0.0)	0	(0.0)	93	(90.3)
Education – n (% ^a^)								
University degree	16,482	(13.0)	7,437	(12.6)	8,994	(13.4)	51	(18.3)
Country of birth – *n* (%^a^)								
Sweden	79,751	(84.3)	38,752	(84.5)	40,793	(84.1)	206	(75.2)
Born outside of Sweden	7,705	(15.7)	3,526	(15.5)	4,133	(15.9)	46	(24.8)
Urbanicity – n (% ^a^)								
Larger city	19,299	(28.4)	8,989	(27.5)	10,245	(29.2)	65	(38.7)
Smaller city	25,114	(32.8)	12,090	(32.6)	12,955	(33.0)	69	(28.3)
Rural community	43,043	(38.8)	21,199	(39.9)	21,726	(37.8)	118	(33.0)
Sever physical impairment – *n* (%^a^)								
No sever physical impairment	74,169	(88.9)	35,827	(88.6)	38,138	(89.1)	204	(88.0)
Sever physical impairment	9,278	(11.1)	4,679	(11.4)	4,565	(10.9)	34	(12.0)

^a^ Weighted percentage

**Table 2:** Sociodemographic distribution by gender modality in the Swedish National public health survey (2020, 2021, and 2022) among participants 65 years or older

	Heterosexual, *n* = 81,881		Bisexual, *n* = 458		Lesbian/gay, *n* = 306		Bisexual vs. heterosexual	Lesbian/gay vs. heterosexual
							Sig.	Sig.
Mean age – years (SD)	74.1	(6.1)	73.6	(6.2)	71.9	(5.2)	*p* <.117	*p* <.001
Education – n (% ^a^)							*p* <.001	*p* <.001
University degree	16,054	(13.4)	135	(24.3)	117	(28.7)		
Country of birth – *n* (%^a^)							*p* <.001	*p* <.001
Sweden	75,069	(85.4)	369	(70.2)	268	(74.5)		
Born outside of Sweden	6,812	(14.6)	89	(29.8)	38	(25.5)		
Urbanicity – n (% ^a^)							*p* =.002	*p* <.001
Larger city	18,268	(28.5)	139	(39.0)	124	(52.3)		
Smaller city	23,593	(32.9)	107	(24.4)	82	(32.0)		
Rural community	40,020	(38.6)	212	(36.6)	100	(15.7)		
Sever physical impairment – *n* (%^a^)							*p* =.850	*p* =.208
No sever physical impairment	69,794	(88.9)	373	(89.3)	251	(92.2)		
Sever physical impairment	8,586	(11.1)	53	(10.7)	36	(7.8)		

^a^ Weighted percentage

### Sexual and gender minority differences in health outcome variables

Sexual orientation differences in self-reported health, physical health symptoms, psychological distress, and positive mental health are presented separately for men and women in Table 3. Among men, the only significant difference in health outcomes was a higher reported frequency of psychological distress among gay men compared to heterosexual men (*d* =−0.21). Among women, the only significant difference in health outcomes was a lower reported experience of positive well-being among lesbian women compared to heterosexual women (*d* =−0.44).

**Table 3:** Physical health symptoms, psychological distress, and positive well-being among participants 65 years or older by sexual orientation and gender minority status

	Men						Difference between bisexual and heterosexual		Difference between gay men and heterosexual	
	Heterosexual, *n*=39,513		Bisexual, *n*=283		Gay, *n*=194					
Health outcome variable							Sig.	Odds Ratio (OR) or Cohen’s *d with 95% CI*	Sig.	Odds Ratio (OR) or Cohen’s *d with 95% CI*
Poor self-rated health – percent (95% CI)	37.9	(37.2, 38.9)	33.5	(24.7, 42.2)	41.5	(31.7, 51.3)	*p* =.336	OR =.82 (.56, 1.22)	*p* =.185	OR = 1.31 (.88, 1.96)
Physical health symptoms – mean (SD)	0.62	(1.05)	0.60	(0.91)	0.53	(0.91)	*p* =.860	*d* =.02 (-.16,.20)	*p* =.379	*d* =.09 (-.11,.28)
Psychological distress – mean (SD)	3.78	(4.00)	3.92	(3.95)	4.61	(4.69)	*p* =.711	*d* =-.04 (-.22,.15)	*p* =.037	*d* =-.21 (-.40,.01)
Positive well-being – mean (SD)	21.60	(3.81)	21.71	(4.52)	20.94	(4.54)	*p* =.775	*d* =-.03 (-.22,.16)	*p* =.089	*d* =.17 (-.03,.37)

	Women									
	Heterosexual, *n*=42,345		Bisexual, *n*=167		Lesbian, *n*=109		Difference between bisexual and heterosexual		Difference between lesbian women and heterosexual	
Health outcome variable							Sig.	Odds Ratio (OR) or Cohen’s *d with 95% CI*	Sig.	Odds Ratio (OR) or Cohen’s *d with 95% CI*
Poor self-rated health – percent (95% CI)	40.6	(39.9, 41.4)	47.4	(36.3, 58.4)	48.1	(33.4, 62.8)	*p* =.151	OR = 1.38 (.89, 2.15)	*p* =.148	OR = 1.53 (.86, 2.72)
Physical health symptoms – mean (SD)	0.86	(1.27)	1.11	(1.37)	1.13	(1.45)	*p* =.067	*d* =-.20 (-.42,.01)	*p* =.139	*d* =-.21 (-.50,.07)
Psychological distress – mean (SD)	4.47	(4.32)	5.32	(4.66)	5.39	(4.81)	*p* =.074	*d* =-.20 (-.41,.02)	*p* =.151	*d* =-.21 (-.50,.08)
Positive well-being – mean (SD)	21.26	(3.87)	20.54	(3.77)	19.56	(3.96)	*p* =.096	*d* =.19 (-.03,.41)	*p* =.003	*d* =-.44 (.15,.73)

	Gender minority status							
	Cisgender, *n*=87,204			Gender minorities, *n*=252			Difference between gender minorities and cisgender	
Health outcome variable							Sig.	Odds Ratio (OR) or Cohen’s *d with 95% CI*
Poor self-rated health – percent (95% CI)	40.0	(39.4, 40.5)		29.4		(20.4, 38.4)	*p* =.060	OR =.66 (.43, 1.02)
Physical health symptoms – mean (SD)	0.75	(1.18)		0.59		(1.09)	*p* =.180	*d* =.13 (-.06,.32)
Psychological distress – mean (SD)	4.26	(4.32)		4.96		(5.19)	*p* =.095	*d* =-.16 (-.36,.03)
Positive well-being – mean (SD)	21.38	(3.91)		21.83		(3.82)	*p* =.286	*d* =-.12 (-.33,.06)

Note: Weighted proportions and means.

Difference between gender minorities and cisgender individuals on the health outcome variables are also reported in Table 3. There were no significant differences in health outcome between gender minorities and cisgender individuals.

### Sexual and gender minority differences in theory-derived determinants of health

Sexual orientation differences in interpersonal stress, social connectedness, and economic hardship are presented separately for men and women in Table 4.

**Table 4:** Interpersonal stress, social connectedness, and economic hardship among participants 65 years or older by sexual orientation and gender minority status

	Men				
	Heterosexual, *n* = 39,513	Bisexual men, *n* = 283	Gay men, *n* = 194	Bisexual vs. heterosexual	Gay men vs. heterosexual
	**%** ^a^	**%** ^a^	**%** ^a^	*Sig.*	*Sig.*
Interpersonal stress:					
Discrimination	7.2	11.9	20.8	*p* =.053	*p* <.001
Victimization or threats	3.0	9.7	5.1	*p* <.001	*p* =.235
Lack of social trust	17.8	31.4	13.7	*p* <.001	*p* =.280
Social connectedness:					
Living alone	23.2	50.0	52.5	*p* <.001	*p* <.001
Lack of social support	13.0	29.1	21.8	*p* <.001	*p* =.009
Low social participation	27.7	25.4	15.8	*p* =.586	*p* =.008
Economic hardship:					
Low household income	14.1	30.5	31.0	*p* <.001	*p* <.001
Financial stress	4.5	9.5	12.9	*p* =.011	*p* <.001

	Women				
	Heterosexual, *n* = 42,345	Bisexual, *n* = 167	Lesbian/gay, *n* = 109	Bisexual vs. heterosexual	Lesbian vs. heterosexual
	**%** ^a^	**%** ^a^	**%** ^a^		
Interpersonal stress:					
Discrimination	9.5	14.5	22.9	*p* =.121	*p* =.002
Victimization or threats	2.0	2.4	2.1	*p* =.805	*p* =.978
Lack of social trust	18.3	26.5	6.5	*p* =.054	*p* =.039
Social connectedness:					
Living alone	40.5	60.2	44.7	*p* <.001	*p* =.561
Lack of social support	10.7	27.7	18.8	*p* <.001	*p* =.071
Low social participation	26.8	34.9	25.0	*p* =.093	*p* =.783
Economic hardship:					
Low household income	27.9	43.4	35.4	*p* =.002	*p* =.245
Financial stress	5.2	16.9	16.7	*p* <.001	*p* <.001

	Gender minority status				
	Cisgender, *n* = 87,204		Gender minorities, *n* = 252	Gender minorities vs. cisgender	
	**%** ^a^		**%** ^a^		
Interpersonal stress:					
Discrimination	8,4		6,7	*p* =.535	
Victimization or threats	2,5		14,6	*p* <.001	
Lack of social trust	19,0		32,7	*p* <.001	
Social connectedness:					
Living alone	33,0		36,2	*p* =.481	
Lack of social support	12,9		23,1	*p* =.002	
Low social participation	28,4		29,5	*p* =.798	
Economic hardship:					
Low household income	22,5		19,0	*p* =.402	
Financial stress	5,5		8,6	*p* =.160	

^a^ Weighted proportion

Among men, bisexuals reported more often past 12-month victimization (χ^2^ = 17.19, *df* = 1, *P* <.001), and they were more likely to report lack of social trust (χ^2^ = 14.56, *df* = 1, *P* <.001). Bisexual men were much more likely to be living alone (χ^2^ = 46.98, *df* = 1, *P* <.001), reporting lack of social support (χ^2^ = 6.15, *df* = 1, *P* <.001), low household income (χ^2^ = 25.74, *df* = 1, *P* <.001), and experiencing financial stress (χ^2^ = 6.43, *df* = 1, *P* =.011). Gay men were more likely to report past 3 months experience of discrimination (χ^2^ = 27.26, *df* = 1, *P* <.001). Gay men were also more likely to be living alone (χ^2^ = 48.05, *df* = 1, *P* <.001), reporting a lack of social support (χ^2^ = 6.76, *df* = 1, *P* =.009), low household income (χ^2^ = 23.19, *df* = 1, *P* <.001), and to experience financial stress (χ^2^ = 15.88, *df* = 1, *P* <.001). Gay men reported greater social participation than heterosexual men (χ^2^ = 7.03, *df* = 1, *P* =.008).

Among women, bisexuals were significantly more likely to be living alone (χ^2^ = 13.33, *df* = 1, *P* <.001), reporting lack of social support (χ^2^ = 24.88, *df* = 1, *P* <.001), low household income (χ^2^ = 9.85, *df* = 1, *P* =.002), and experiencing financial stress (χ^2^ = 22.24, *df* = 1, *P* <.001). Lesbian women were more likely to report past 3-months experience of discrimination (χ^2^ = 10.07, *df* = 1, *P* =.002) and experiencing financial stress (χ^2^ = 12.50, *df* = 1, *P* <.001). Lesbian women were less likely to report lack of social trust than heterosexual women (χ^2^ = 4.27, *df* = 1, *P* =.039).

Gender minority individuals were more likely to report past 12-month victimization (χ^2^ = 59.15, *df* = 1, *P* <.001), lack of social trust (χ^2^ = 12.68, *df* = 1, *P* <.001), and lack of social support (χ^2^ = 9.56, *df* = 1, *P* =.002).

### Differences in sexual and gender disparities by level of education, migration background, geographic location, and physical disability

Our analysis of sexual orientation and gender minority status disparities by intersecting variables based on level of education, migration background, geographic location (i.e., large city; medium/small town; rural area), and physical disability on our health outcomes showed a few significant differences. Lesbian women and gay men with higher education reported lower physical health symptoms (mean = 0.39, SD = 0.87) compared to heterosexuals with high education (mean = 0.67, SD = 1.13). Similarly, lesbian women and gay men born outside of Sweden reported fewer physical health symptoms (mean = 0.34, SD = 0.83), as compared to heterosexuals born outside of Sweden (mean = 0.91, SD = 1.34). Bisexual living in larger cities reported a higher number of physical symptoms (mean = 1.08, SD = 1.19) and lower positive well-being (mean = 19.81, SD = 3.87) than heterosexuals living in larger cities (mean_physical health symtpoms_ = 0.73, SD = 1.17; mean_positive well−being_ = 21.43, SD = 3.95).

Among gender minorities, gender minorities born outside of Sweden reported lower frequency of physical symptoms (mean = 0.31, SD = 0.99), but greater psychological distress (mean = 8.00, SD = 7.26) compared to gender minorities born in Sweden (mean_physical health symtpoms_ = 0.68, SD = 1.12; mean_psychological distress_ = 3.94, SD = 3.83).

There was no significant pattern of effect modification based on intersecting difference for any of the other variables.

### Supplementary sexual and gender minority subgroup analyses in health and well-being

Results of analyses comparing differences among lesbian, gay, and bisexual participants, as well as differences between sexual minority and trans participants are presented in Supplementary Table S1. There were no significant sexual and gender minority sub-group differences on any of our health and well-being outcomes.

## Discussion

This study provides one of the most comprehensive population-based examinations of health disparities among older SGM adults outside the United States. Contrary to expectations based on minority stress theory, we found only modest differences in health outcomes between SGM and heterosexual cisgender older adults. These findings challenge assumptions of uniformly poorer health among older SGM individuals and suggest the presence of resilience factors that may buffer the effects of social adversity.

Our results align with emerging sexual minority health perspectives such as strength-based models and distinct lifeways theory, which posits that SGM individuals navigate unique social roles and networks that may both pose risks and offer protective benefits [31, 52]. For example, while SGM participants were more likely to live alone and report financial stress, they also demonstrated higher educational attainment and, in some cases, greater social participation. These findings underscore the importance of considering both structural disadvantages and community-based strengths in understanding health trajectories among older SGM adults.

Subgroup analyses revealed that disparities were not uniform across all SGM individuals. For instance, bisexual individuals and those living in urban areas reported more physical symptoms and lower well-being, while foreign-born SGM individuals exhibited a paradoxical pattern of fewer physical symptoms but greater psychological distress. These nuanced patterns highlight the need for intersectional approaches that account for the interplay of sexual orientation, gender identity, socioeconomic status, migration background, and geography when studying older SGM individuals’ health and well-being. The importance of subgroups differences is also supported by findings from previous studies [53–56].

Importantly, our findings suggest that the social determinants of health – particularly interpersonal stress and economic hardship – remain significant challenges for older SGM adults. These stressors may not always manifest as immediate health outcomes but could have cumulative effects over time. Interventions aimed at reducing discrimination, enhancing social support, and addressing economic insecurity are critical to promoting health equity in aging populations.

Overall, our findings are consistent with prior research documenting both disparities and resilience among older SGM adults. Studies have shown that older SGM individuals often face elevated risks of psychological distress, social isolation, and economic hardship due to cumulative exposure to stigma and discrimination across the life course [41, 44]. For example, Fredriksen-Goldsen et al. (2011) found that older SGM adults in the U.S. were more likely to experience poor general health, disability, and mental distress compared to heterosexual peers [41]. Similarly, Yarns et al. (2016) highlighted the mental health vulnerabilities of older SGM adults, emphasizing the role of minority stress [44]. However, other studies have emphasized the resilience of these populations, pointing to the protective effects of chosen families, community engagement, and adaptive coping strategies developed over time [35, 37, 42, 57, 58]. Our findings echo this dual narrative: while older SGM adults in Sweden report higher levels of social and economic stressors, these do not consistently translate into poorer health outcomes. This may reflect both individual-level resilience and the buffering effects of Sweden’s relatively inclusive social policies and welfare systems [59]. Moreover, our results support the distinct lifeways theory, which suggests that SGM individuals’ unique social trajectories — such as higher educational attainment and alternative support networks — can shape both risk and resilience in later life [31].

A notable strength of the current study is the inclusion of a relatively large group of gender minority people. This is, to our knowledge, the first population-based study specifically focused on the health and well-being of older gender minority people. Gender minorities face distinct and multifaceted health challenges that are shaped by a complex interplay of social, structural, and individual-level factors [25]. Many recent studies, although mostly limited by non-representative samples, have consistently documented poorer health and well-being among gender minorities [60]. In light of these findings, it is particularly noteworthy that we did not find evidence of increased risks of poor mental health and well-being among older gender minorities in the current study. Among the health determinants assessed in the current study, we did not have access to several factors that we know are important for the health and well-being of gender minorities. For example, structural barriers in healthcare access, misgendering, and lack of provider competence regarding gender-affirming care [23–25]. These experiences require attention in future studies.

Several limitations should be considered when interpreting the findings of this study. First, the cross-sectional design precludes any conclusions about causality or the directionality of associations between sexual and gender minority status, social determinants, and health outcomes. Longitudinal studies are needed to better understand how these relationships evolve over time and across different stages of aging. Second, although the sample was large and population-based, the number of respondents identifying as sexual or gender minorities — particularly bisexual men, lesbian women, and gender minorities — was relatively small. This limited statistical power to detect differences in some subgroup analyses and may have contributed to the modest effects observed. Third, the measures of sexual orientation and gender identity were based on self-report and limited response options, which may not fully capture the diversity and fluidity of identities among older adults. Additionally, the survey did not include any SGM specific questions, e.g., questions about degree of openness with identity, connectedness with SGM communities, information on lifetime experiences of stigma, internalized homophobia or transphobia, or access to affirming healthcare, which are important factors in understanding health disparities. Finally, while Sweden’s inclusive social policies may buffer some of the negative effects of minority stress, these findings may not generalize to countries with different sociopolitical contexts or healthcare systems.

In conclusion, this study contributes to a more differentiated understanding of aging among SGM populations. It calls for a shift from deficit-based models toward frameworks that recognize both vulnerability and resilience. Future research should explore longitudinal patterns and mechanisms of adaptation, including assessment of strengthening factors and resilience factors in addition to the stigma-based challenges that sexual and gender minorities are disproportionately exposed to, as well as culturally tailored interventions that support healthy aging in diverse communities.

## Supplementary Information

Supplementary Material 1.

## Data Availability

All data were produced under the Swedish Statistics Act and the European Union Data Protection Regulation, according to which privacy concerns restrict the availability of personal data for research. Aggregated data can be made available by the authors, subject to ethical vetting. Code can be made available through contact with the corresponding author. Correspondence concerning the article should be addressed: Richard Bränström, PhD, Division of Psychology, Department of Clinical Neuroscience, Karolinska Institutet, Nobels väg 9, 171 77 Stockholm, Sweden Tel: +46 768 111 721. E-mail: [richard.branstrom@ki.se](mailto: richard.branstrom@ki.se).
