# Supplementary material for: Resilience and distinct lifeways: sexual orientation and gender minority status differences in health, well-being, and social determinants of health in a population-based sample of older adults
Source: BMC Public Health. 2025 Dec 23;25:4276. doi: 10.1186/s12889-025-25426-w (PMC12723854; doi:10.1186/s12889-025-25426-w)
Supplement: Supplementary file 1 — Supplementary Material 1. [file 12889_2025_25426_MOESM1_ESM.docx]

**Supplementary Table S1**

Physical health symptoms, psychological distress, and positive well-being among participants 65 years or older with comparisons between specific sexual orientation groups and between gender minority as compared to LGB cisgender individuals.

|  | Men | | | | | | | | |  |  | |
| --- | --- | --- | --- | --- | --- | --- | --- | --- | --- | --- | --- | --- |
|  | Heterosexual, *n*=39,513 | | | Bisexual men, *n*=283 | | | Gay men,  *n*=194 | | |  | Difference between gay men and bisexuals | |
| **Health outcome variable** |  | | |  | | |  | | |  | Sig. | Odds Ratio (OR) or Cohen’s *d  with 95% CI* |
| Poor self-rated health – percent (95% CI) | 37.9 | (37.2, 38.9) | | 33.5 | | (24.7, 42.2) | 41.5 | | (31.7, 51.3) |  | *p* = .226 | OR = 1.41 (.81, 2.46) |
| Physical health symptoms – mean (SD) | 0.62 | (1.05) | | 0.60 | | (0.91) | 0.53 | | (0.91) |  | *p* = .545 | *-* |
| Psychological distress – mean (SD) | 3.78 | (4.00) | | 3.92 | | (3.95) | 4.61 | | (4.69) |  | *p* = .593 | *-* |
| Positive well-being – mean (SD) | 21.60 | (3.81) | | 21.71 | | (4.52) | 20.94 | | (4.54) |  | *p* = .229 | *-* |
|  |  |  | |  | |  |  | |  |  |  |  |
|  | Women | | | | | | | | |  |  | |
|  | Heterosexual, *n*=42,345 | | | Bisexual,  *n*=167 | | | Lesbian/gay,  *n*=109 | | |  | Difference between lesbian women and bisexuals | |
| **Health outcome variable** |  | | |  | | |  | | |  | Sig. | Odds Ratio (OR)  or Cohen’s *d  with 95% CI* |
| Poor self-rated health – percent (95% CI) | 40.6 | (39.9, 41.4) | | 47.4 | | (36.3, 58.4) | 48.1 | | (33.4, 62.8) |  | *p* = .939 | OR = 1.03 (.50, 2.10) |
| Physical health symptoms – mean (SD) | 0.86 | (1.27) | | 1.11 | | (1.37) | 1.13 | | (1.45) |  | *p* = .949 | *-* |
| Psychological distress – mean (SD) | 4.47 | (4.32) | | 5.32 | | (4.66) | 5.39 | | (4.81) |  | *p* = .778 | *-* |
| Positive well-being – mean (SD) | 21.26 | (3.87) | | 20.54 | | (3.77) | 19.56 | | (3.96) |  | *p* = .174 | *-* |
|  |  |  | |  | |  |  | |  |  |  |  |
|  | Gender minority status | | | | | | | | |  |  | |
|  | Cisgender LGB individuals,  *n*=741 | | | | Gender minorities,  *n*=252 | | | | |  | Difference between gender minorities and cisgender | |
| **Health outcome variable** |  | | | |  | | | | |  | Sig. | Odds Ratio (OR)  or Cohen’s *d  with 95% CI* |
| Poor self-rated health – percent (95% CI) | 40.0 | | (39.4, 40.5) | | 29.4 | | | (20.4, 38.4) | |  | *p* = .202 | OR = .82 (.61, 1.11) |
| Physical health symptoms – mean (SD) | 0.77 | | (1.15) | | 0.59 | | | (1.09) | |  | *p* = .148 | *-* |
| Psychological distress – mean (SD) | 4.71 | | (4.49) | | 4.96 | | | (5.19) | |  | *p* = .628 | *-* |
| Positive well-being – mean (SD) | 20.88 | | (4.29) | | 21.83 | | | (3.82) | |  | *p* = .063 | *-* |
| Note: Weighted proportions and means. | | | | | | | | | | | | |
